# The Effect of Foliar Spraying of Different Selenium Fertilizers on the Growth, Yield, and Quality of Garlic (*Allium sativum* L.)

**DOI:** 10.3390/plants14162505

**Published:** 2025-08-12

**Authors:** Guangyang Liu, Jie Ge, Jide Fan, Yongqiang Zhao, Xinjuan Lu, Canyu Liu, Biwei Zhang, Qingqing Yang, Mengqian Li, Yan Yang, Yi Feng, Feng Yang

**Affiliations:** 1 Key Laboratory of Biology and Genetic Breeding of Sweetpotato, Ministry of Agriculture and Rural Affairs, Xuzhou Institute of Agricultural Sciences in Jiangsu Xuhuai Area, Xuzhou 221131, China; 2020104114@stu.njau.edu.cn (G.L.); 20201017@jaas.ac.cn (J.G.); 20071002@jaas.ac.cn (J.F.); 20091004@jaas.ac.cn (Y.Z.); 20081004@jaas.ac.cn (X.L.); 20161005@jaas.ac.cn (C.L.); 20181004@jaas.ac.cn (B.Z.); 20201006@jaas.ac.cn (Q.Y.); limengqian@jaas.ac.cn (M.L.); 20230064@jaas.ac.cn (Y.Y.); 821012450338@caas.cn (Y.F.); 2 Sweetpotato Research Institute, Chinese Academy of Agricultural Sciences, Xuzhou 221131, China

**Keywords:** garlic (*Allium sativum* L.), selenium enrichment, nano-selenium fertilizer, membership function, comprehensive evaluation

## Abstract

This study investigated the effects of four selenium fertilizers (nano-Se, EDTA-chelated Se, organic Se, and microbial Se) at three concentrations (50, 25, and 12.5 mg·L^−1^) on garlic (*Allium sativum* L. cv. ‘Xusuan 918’) through foliar application during critical growth stages. Comprehensive evaluation combining agronomic traits, yield components, nutritional quality (soluble sugars, vitamin C), and selenium accumulation revealed distinct fertilizer-specific responses. Organic Se at 50 mg·L^−1^ (O1) maximized vegetative growth (21.83% increased plant spread), while 25 mg·L^−1^ microbial Se (M2) showed optimal yield enhancement (10.04% higher bulb dry weight vs. CK). Notably, 50 mg·L^−1^ nano-Se (N1) achieved simultaneous improvement in nutritional quality and selenium biofortification (29-fold bulb Se enrichment). Principal component analysis integrated with membership function method identified N1 treatment (D-value = 0.571) as the most effective protocol for selenium-enriched garlic production, demonstrating the importance of fertilizer selection for crop-specific selenium management strategies.

## 1. Introduction

Garlic (*Allium sativum* L.), a biennial herb of the genus Allium in the Liliaceae family, holds significant economic and medicinal value. Its active component, allicin, is hailed as a “natural broad-spectrum antibiotic,” while its precursor, alliin, is referred to as “penicillin grown from the earth” [1]. China, as the world’s leading garlic producer, accounts for over 90% of global output. For instance, in Jiangsu Province, China, the garlic planting area reached 82,000 hectares in 2024 (an increase of 3333.33 hectares), with a production of 1.42 million tons (up by 70,000 tons), and export earnings of 420 million US dollars [2] highlighting the crucial role of this industry in increasing farmers’ income in China.

Selenium, an essential trace element for the human body, participates in various metabolic processes. Its deficiency can lead to multiple diseases [3]. Clinical studies have confirmed that appropriate selenium supplementation can effectively alleviate lead poisoning-induced kidney damage in children (*p* < 0.05), reduce the side effects of radiotherapy, and potentially improve epilepsy symptoms [4,5,6]. Selenium deficiency is a significant issue in China, with 72% of the population residing in selenium-deficient areas. Additionally, soil selenium deficiency problems are present in 22 provinces across the country [7]. Soil selenium deficiency reduces the ability of plants to absorb and utilize selenium, resulting in generally low selenium content in agricultural products [8]. Garlic exhibits a remarkable selenium enrichment capacity, with its selenium content reaching 20–30 times that of ordinary vegetables [9,10], making it an ideal dietary selenium supplement carrier [11].

Previous studies have shown that providing trace elements such as selenium to plants through agronomic and biological fortification methods can improve the nutritional quality of crops and increase the content of trace elements in products and by-products [12,13,14]. Therefore, the rational application of selenium fertilizers is particularly crucial when growing garlic in selenium-deficient areas. The application of selenium fertilizers not only increases the selenium content in crops but also enhances their yield, quality, and stress resistance: during the study of wheat, it was found that selenium fertilizer can significantly increase the selenium content in grains and enhance stress resistance [15]; and during the study of millet, it was confirmed that selenium fertilizers could regulate plant metabolic characteristics, thereby increasing protein content and reducing fat content [16]. Currently, selenium fertilizers are mainly classified into four types: nano-selenium fertilizers, biological selenium fertilizers (microbial selenium fertilizers), organic selenium fertilizers, and inorganic selenium fertilizers [17]. There are relatively few studies on the absorption and accumulation of selenium in crop organs, and its efficiency is significantly affected by the method of selenium application. Multiple studies have confirmed that foliar spraying is more effective than soil application [18], as foliar spraying can quickly transport nutrients to specific parts of the plant, thereby optimizing nutrient absorption and growth and development [19]. The absorption efficiency of different selenium fertilizers shows a gradient difference: for example, in rice, nano-selenium fertilizers > organic selenium fertilizers > inorganic selenium fertilizers [20,21,22]. Among them, it was found that compared with selenite, high-dose nano-selenium fertilizers have stronger antioxidant effects and lower toxicity [23]. The concentration of selenium application must be strictly controlled: at high concentrations, selenium may be toxic to some plants, leading to growth retardation, changes in photosynthetic pigments, and chlorosis [24]. In previous foliar spraying and soil application experiments, the selenium concentration was all below 100 g of selenium per hectare, and within this range, the promoting effect of selenium on plant growth was observed [25,26].

The evaluation of selenium fertilizer efficacy requires a multidimensional assessment system to address the limitations of single-indicator approaches. As evidenced by previous studies [27], the combined application of principal component analysis and membership function methodology has proven effective in fertilizer evaluation research. This integrated assessment framework significantly enhances both the scientific validity and accuracy of conclusions regarding selenium fertilizer applications.

Current research on garlic mainly focuses on its stress resistance, reproductive characteristics, and biofortification [28,29,30,31,32]. Among the studies on biofortification, research on fertilization techniques for selenium-enriched garlic production is relatively scarce. Previous studies on foliar application of sulfur fertilizer to garlic were conducted after the garlic regreening period (around 20 March), with applications every seven days until the bulb expansion period, which provides a reference for setting the fertilization time in this study [32]. In this study, four distinct selenium fertilizer formulations were systematically evaluated: nano-selenium fertilizer, EDTA-chelated selenium fertilizer, organic selenium fertilizer, and microbial selenium fertilizer. Using ‘Xusuan 918’ garlic as the experimental material, we employed comprehensive analytical approaches, including principal component analysis and membership function methodology, to identify the optimal fertilization protocol for selenium-enriched garlic cultivation.

## 2. Results

### 2.1. Growth Status of Plant Shoots Post-Fertilization Treatment

Table 1 data show that different treatments significantly affect aboveground growth indicators in garlic. The analysis of growth parameters revealed significant variations among treatments. Regarding plant height, the E3 treatment demonstrated superior performance with an average height of 67.65 cm, representing a 12.00% significant increase compared to the control (CK) (*p* < 0.05). Notably, only the N2 treatment showed marginally lower values than CK, though this difference was not statistically significant. For plant width measurements, all fertilization treatments except O2 exhibited significant enhancement over CK, with O1 showing the most pronounced improvement (25.17%), followed by N1 (21.84%). Leaf morphological analysis indicated that all treatments except O2 and E2 significantly exceeded CK in leaf length, particularly O1, which achieved an 18.50% increase. In terms of leaf width, only the O1 treatment showed significant superiority to CK, while other treatment groups maintained comparable dimensions to the control. All treatment groups had seven functional leaves, with no statistically significant difference. For aboveground pseudostem traits: all treatments except E1 outperformed CK, with O1 having the tallest pseudostem height (38.93 cm); all fertilization treatments had significantly thicker pseudostems than CK, particularly the O2 treatment (19.48 mm). Comprehensive analysis demonstrates that the O1 treatment yields optimal efficacy in promoting garlic shoot growth and development.

### 2.2. Effect of Selenium Fertilizer Application on Garlic Bulb Yield

The data in Table 2 shows yield-related traits of garlic bulbs. In terms of fresh weight, the microbial selenium fertilizer treatments showed the best yield-increasing effect; all three treatments achieved fresh weight yields exceeding 27 tons per hectare (t ha^−1^), and all were significantly higher than CK. Among them, the M2 treatment had the best yield increase effect, increasing yield by 17.78% compared to CK. Yield analysis revealed distinct treatment effects. The O2 treatment exhibited the most limited yield enhancement, with a marginal increase of merely 0.71 t ha^−1^. Microbial selenium fertilizer treatments consistently demonstrated superior performance in dry weight production, with all three replicates exceeding 20 t ha^−1^. Particularly, the M2 treatment showed the most pronounced yield improvement, registering a significant 18.75% increase over CK (*p* < 0.05), surpassing its corresponding fresh weight gain. The trial-wide average dry-to-fresh weight ratio was 75.46%, with CK measuring 73.18%. All treatment groups exceeded the control in this parameter, with N2 achieving the highest ratio at 79.91%.

Morphological examination showed contrasting patterns between bulb traits. For bulb height, only the N1 treatment significantly surpassed CK, while other treatments remained comparable. Conversely, all treatments demonstrated significantly increased bulb diameters relative to CK, with most exceeding 60 cm except N2. As single-bulb weight directly correlates with yield, these dimensional improvements have important agronomic implications. The three microbial selenium fertilizer treatment groups all achieved single bulb weights above 69 g, with the M2 treatment reaching 71.53 g, an increase of 10.90 g compared to CK. Among all treatment groups, only the N2 treatment showed no significant difference in single bulb weight compared to CK; similarly, the fresh weight and dry weight of this treatment also showed no significant difference from CK.

### 2.3. Effects of Different Selenium Fertilizer Treatments on Garlic Bulb Nutritional Components

The data in Table 3 show the relevant content of nutrients in garlic bulbs. In terms of allicin content, the application of nano-selenium fertilizer and organic selenium fertilizer had the most significant promoting effect. The allicin content in all six treatment groups of these two selenium fertilizers was significantly higher than CK. N1 had the highest allicin content, reaching 2.70 mg g^−1^, representing a 12.03% increase compared to CK. Regarding the vitamin C (Vc) content indicator, only the application of nano-selenium fertilizer significantly increased Vc content in garlic bulbs; applications of other types of selenium fertilizer showed no significant difference. Among them, the N1 treatment group had the highest Vc content, reaching 0.93 mg g^−1^. In terms of total soluble sugar (TSS) content, except for the M3 treatment, the TSS content in garlic bulbs of all other treatments was significantly higher than the CK group. The O1 treatment showed the highest TSS content, reaching 19.46%. Except for the E3 treatment, the soluble protein content in garlic bulbs of all other treatment groups was higher than CK. The O1 treatment showed the largest increase, rising by 5.89%.

### 2.4. Selenium Accumulation in Garlic Under Different Treatments

The data in Table 4 show the selenium content in garlic leaves and bulbs under different selenium application treatments. Experimental results indicate that the selenium enrichment efficiency in garlic varied significantly among selenium fertilizer treatments (*p* < 0.05). Regarding selenium content in leaves, the nano-selenium and organic selenium treatment groups exhibited the most prominent effects. Specifically, the N1 treatment increased leaf selenium content by 25-fold compared to the control (CK), while the microbial selenium (M3) treatment group showed the smallest increase (only 3-fold). The trend in bulb selenium content aligned with that in leaves: the N1 treatment achieved the highest accumulation efficiency (29-fold increase versus CK), and the M3 group showed the lowest increase (3-fold). Notably, calculations of the bulb/leaf selenium content ratio revealed that M1, M2, and E1 treatments had the highest translocation efficiency (ratio ≥ 0.55), indicating their effectiveness in promoting selenium transport from vegetative organs to storage organs. These results provide critical insights for selecting selenium fertilizers in selenium-enriched garlic production.

### 2.5. Comprehensive Evaluation of Fertilization Efficacy Under Different Treatments

**Figure 1:**
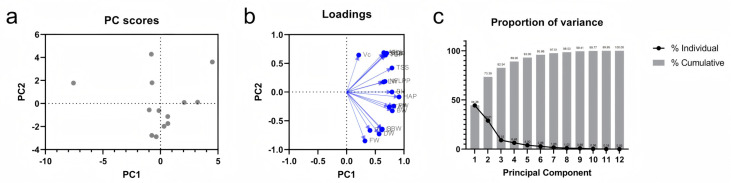
Principal component analysis of 18 indices in garlic under different selenium application treatments: (**a**), PC Scores, each point represents a row of the original data table. (**b**), Loadings, principal component loadings reflect the correlation between variables and principal components; FW, fresh weight; DW, dry weight; PH, plant height; PW, plant width; LL, leaf length; LW, leaf width; NFLPP, number of functional leaves per plant; HAP, height of above-ground pseudostem; TAP, thickness of above-ground pseudostem; BH, bulb height; BW, bulb width; SBW, single bulb weight; Vc, vitamin C; TSS, total soluble sugar; TSP, total soluble protein; SCL, selenium content in leaves; SCB, selenium content in bulbs. (**c**), proportion of eigenvalue.

Due to significant correlations among the indicators, resulting in overlapping information, principal component analysis (PCA) was performed to minimize redundancy (Figure 1a). Four principal components were precisely extracted based on eigenvalues greater than one. The contribution rates of these components were 44.379%, 29.007%, 9.153%, and 6.460% respectively, with a cumulative contribution rate of 88.999% (Figure 1c). This demonstrates that these four independent composite indicators effectively replace the original 18 individual metrics. Loadings of each principal component revealed that PC1 (highest contribution) was primarily composed of aboveground pseudostem height, plant spread, bulb height, and bulb diameter; PC2 mainly comprised fresh weight, dry weight, and plant height; PC3 primarily included leaf width and number of leaves per plant; PC4 chiefly consisted of vitamin C content and single-bulb weight (Table S4).

Based on PCA outcomes, principal component scores for each treatment were calculated using Formula (2). Weights for each principal component were determined via Formula (3), and membership function values (μ) were computed with Formula (4). Subsequently, comprehensive fertilization efficacy scores (D) were derived through Formula (5), where a higher D-value indicates superior efficacy.

Results showed the descending ranking of D-values: N1 → N2 → N3 → O1 → E1 →O3 → O2 → M1 → M2 → E2 → E3 → M3 → CK. Treatments N1, N2, and N3 all exhibited D > 0.5, confirming strong fertilization efficacy. Notably, N1 achieved a significantly higher D-value (0.571) than the other 12 treatments (Table 5).

## 3. Discussion

### 3.1. Application of Fertilizers Promotes the Growth of Garlic Aboveground Parts and Bulbs

In this study, to minimize the influence of randomness, we conducted two consecutive garlic selenium fertilizer screening experiments from 2023 to 2025. During these two years, the weather at the planting site was abnormal (Table S6). The temperature was abnormally high, and there was a continuous drought from March to April in 2024 and 2025. Previous studies have shown that high temperature and drought can affect garlic yield, especially the abnormal weather during the growth stage after garlic regreening, which has a particularly significant impact on the yield of garlic bulbs [30,33]. Plant growth is strongly affected by drought. Water deficiency leads to stomatal closure, photoinhibition, and a decrease in PSII photon yield. Chlorophyll and chloroplast structure accumulate damage [34]. Under water-deficient conditions, leaf photosynthesis decreases initially due to reduced stomatal conductance (triggered by ABA abscisic acid), resulting in reduced CO_2_ absorption. As the stress persists, metabolic damage further inhibits CO_2_ fixation, thereby affecting dry matter accumulation [35]. Many scholars have found that the application of selenate can protect chloroplasts, maintain their structural integrity and functionality, reduce the impact of reactive oxygen species, and improve the stability of electron transfer in photosynthesis, thereby increasing photosynthetic rate and enhancing the drought resistance of plants [36,37]. The garlic variety used in this experiment can achieve a bulb yield of over 24 t ha^−1^ under normal climatic conditions [38]. Previous studies have demonstrated that wheat exposed to unfavorable environmental conditions can result in a significant reduction in yield. However, selenium supplementation has been shown to enhance wheat spike length and increase the content of phenolic compounds in grains [15]. In this study, due to adverse weather conditions, the bulb yield of the control (CK) was only 17.64 t ha^−1^. After applying selenium fertilizer, the maximum dry weight yield could reach 20.95 t ha^−1^ (Table 2), which can reduce the impact of adverse conditions on garlic bulb yield.

Correlation analysis revealed significant associations between aerial growth parameters (leaf length and pseudostem height) and bulb yield (Table S2), suggesting the potential of using vegetative growth indicators as reliable predictors for yield estimation. Notably, treatments N2 and O2 demonstrated inferior performance in promoting aerial growth compared to other concentrations of the same fertilizer types, which consequently resulted in diminished bulb yields. These findings align with previous research [39] demonstrating that low-concentration selenium application (100 g ha^−1^) can enhance dry matter accumulation in plants, highlighting the importance of optimal fertilizer concentration for maximizing crop productivity. In this study, we found that the dry weight ratio of the bulbs in the selenium fertilizer treatment groups was higher than that in the CK group (Table 2), indicating that applying selenium can increase the accumulation of dry matter in garlic bulbs. The reason for this phenomenon may be that applying an appropriate amount of selenium fertilizer can increase the photosynthetic efficiency of plants and produce more photosynthetic products such as total soluble sugar [40,41].

### 3.2. Application of Selenium Fertilizer Can Increase the Selenium Content and Other Nutrient Contents in Garlic Bulbs

In this study, we detected the selenium content in garlic leaves and bulbs and found that the accumulation of selenium in leaves was much higher than that in bulbs. Previous studies have shown that selenium in plants mainly accumulates in the aboveground parts [42]. We can increase the absorption and utilization efficiency of selenium by foliar spraying. By comparing the ratio of selenium content in bulbs and leaves, we found that the transfer of selenium from leaves to bulbs was higher in the treatments with nano-selenium fertilizer, and there was a trend of increasing with concentration (Table 4). This indicates that these treatments can effectively promote the transfer of selenium from vegetative organs to storage organs. This result provides an important basis for the selection of selenium fertilizers in the production of selenium-enriched garlic.

Allicin is a natural compound synthesized by garlic and has protective effects against tissue inflammation and vascular remodeling mediated by oxidative stress [43]. The content of allicin is a core indicator of the nutritional quality of garlic. In our study, we found that the application of nano-selenium fertilizer could significantly increase the allicin content in the bulbs, and the content increased with the increase in nano-selenium fertilizer concentration (Table 3). Through correlation analysis, we discovered that there was a strong correlation between the selenium content and allicin content in garlic bulbs (Table S2), and sulfur plays a key role in the molecular structure of allicin.

Previous studies have shown that selenium and sulfur are both elements in Group VIA, and selenate (SeO_4_^2−^) has similar physicochemical properties to sulfate [44]. A previous study screened a semi-dominant arsenite-tolerant mutant astol1 from a rice mutant library and found that the allelic mutation of this gene could regulate the absorption of sulfur and selenium in rice, enhance sulfur metabolism in rice, promote the synthesis of cysteine and phytochelatins, and achieve multiple effects such as arsenic tolerance in rice, arsenic reduction in rice grains, and sulfur and selenium enrichment [45]. It is generally believed that SeO_4_^2−^ enters plants through sulfate transporters (SULTR). The ability of SULTR to selectively absorb SeO_4_^2−^ and SO_4_^2−^ is related to the external sulfate level, the type of plant, and the type of transporter [46]. These findings provide a new perspective for our next step in studying the mechanism of selenium absorption, transport, and accumulation in garlic from a molecular biology perspective.

When plants are subjected to abiotic stress, the osmotic pressure of cells undergoes significant changes. At this time, plants synthesize substances such as sugars (glucose, fructose, sorbitol, etc.) and amino acids to regulate osmotic pressure and enhance their ability to cope with stress [47]. At the same time, these osmotic adjustment substances also provide defense against reactive oxygen species (ROS) by acting as antioxidants and stabilizing cell structures, proteins, and enzymes [48]. In our study, we found that the content of soluble total sugar and soluble total protein in garlic bulbs was significantly increased compared to the control (CK) (Table 3). This indicates that the application of appropriate selenium fertilizer can improve the stress resistance of garlic and reduce the risk of yield reduction caused by adverse environments. We speculate that applying appropriate selenium fertilizer can improve the stress resistance of garlic and reduce the risk of yield reduction caused by adverse environments. We will further explore the effect of selenium application on garlic stress resistance in future research.

### 3.3. Determining the Appropriate Selenium Application Method Using Principal Component Analysis and Membership Function Method

In this study, we used Pearson correlation analysis to systematically analyze the morphological indicators of the aboveground parts of garlic, bulb-related indicators, quality indicators, and selenium content. The results showed that some indicators, such as dry weight and fresh weight of bulbs, had a highly significant correlation (Figure 1b). To avoid the problem of information overlap caused by the correlation between indicators, we further used principal component analysis (PCA) to reduce the dimensionality of the data [49,50]. At the same time, we introduced the membership function method, a widely used statistical method in comprehensive evaluation of traits [51,52], to construct a comprehensive evaluation value D (integrating the scores of PCA principal components and membership function values) and systematically evaluate the fertilizer efficiency of each selenium application treatment.

Based on the ranking results of D values, all three concentration groups of nano-selenium fertilizer treatment showed significant advantages, among which the 50 mg·L^−1^ nano-selenium fertilizer treatment had the highest D value and was significantly better than other treatment groups (Table 5). In our study, we observed that the D values of O2 (0.479) and O3 (0.482) were very close, indicating that their comprehensive fertilizer efficiencies were almost indistinguishable. Based on this observation, we speculate that the concentration of organic selenium fertilizer in this treatment may lie within the “gentle range” of crop nutrient response. When the fertilizer concentration does not reach the critical threshold required to trigger significant changes in crop growth, the potential improvement in fertilizer efficiency may be buffered by the crop’s inherent compensatory regulatory mechanisms. This buffering effect could explain the reduced apparent differences in fertilizer performance. We believe this finding highlights the importance of understanding crop nutrient response patterns and optimizing fertilizer application strategies accordingly.

As a new selenium source, nano-selenium fertilizer has the advantages of low toxicity and high leaf absorption efficiency compared to traditional selenite fertilizer [53]. In this study, we employed EDTA chelated selenium fertilizer, a novel formulation that offers significant advantages over conventional inorganic selenium fertilizers. This advanced selenium fertilizer forms a stable cyclic complex structure with selenium ions through the use of organic ligands such as EDTA and amino acids. The chelation process exhibits dynamic equilibrium characteristics, enabling selective sequestration of selenium ions by the chelating agents, thereby preventing competitive binding with other metal ions. Furthermore, under physiological conditions in plants, the chelated complexes facilitate sustained selenium release via ligand exchange mechanisms. This unique “capture–release” mechanism enhances selenium uptake efficiency in crops while elevating the safety threshold for application, thereby mitigating the risk of selenium toxicity often associated with traditional inorganic selenium fertilizers [54]. For these reasons, Se-EDTA was selected as the selenium source in this study, aiming to achieve precise regulation and efficient utilization of selenium within plant systems.

This study further confirmed its significant application value in selenium-enriched garlic production through multi-index comprehensive evaluation. It is worth noting that current research on the mechanism of selenium absorption in plants mainly focuses on the absorption process of inorganic selenium by roots, while the absorption and utilization mechanism of nano-selenium fertilizer by leaves is still lacking systematic research [55,56]. In-depth analysis of the absorption and transport mechanism of nano-selenium by leaves is of great theoretical significance for improving the utilization efficiency of selenium fertilizer in garlic, and this is also the focus of our team’s subsequent research.

## 4. Materials and Methods

### 4.1. Plant Materials and Growth Environment

**Figure 2:**
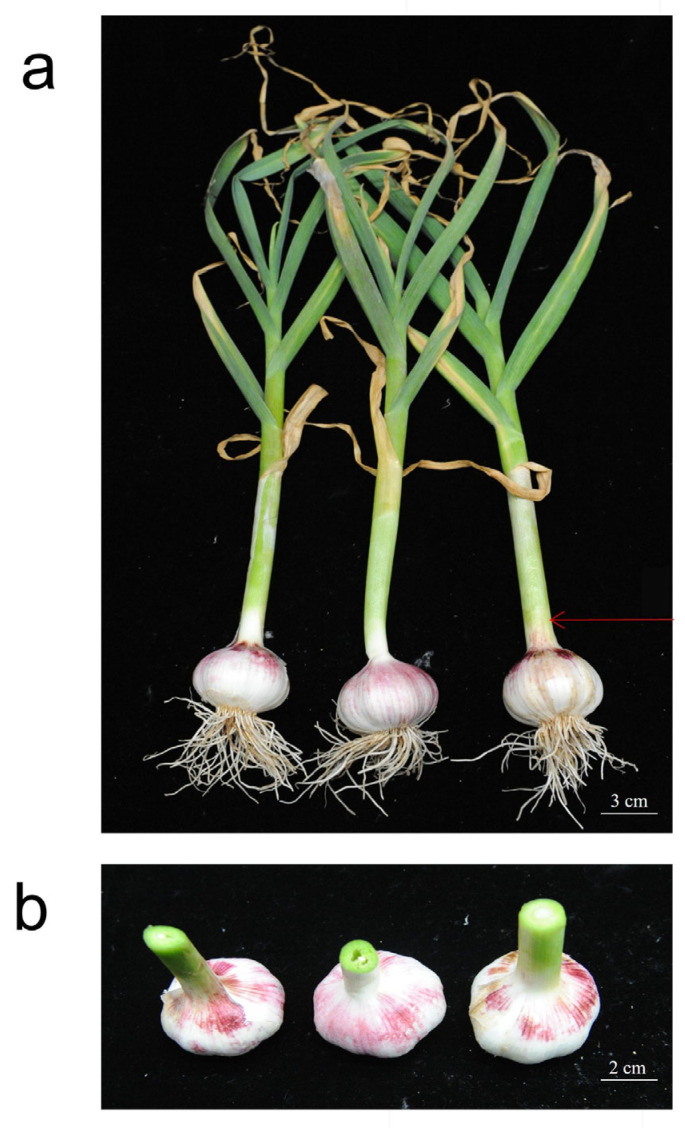
’Xusuan 918’: (**a**), the whole plan’Xusuan918’, the red arrow points to the pseudostem, the white scale indicates a length of 3 cm. (**b**), the garlic bulb after removing the leaves and root system, the white scale indicates a length of 2 cm.

The experimental material was the virus-free garlic variety ‘Xusuan 918’ provided by the Horticulture Research Laboratory of Xuzhou Academy of Agricultural Sciences in Jiangsu Province, China (Figure 2). The basic soil fertility in the experimental area is characterized by the following concentrations: total nitrogen (0.70 g·kg^−1^), available phosphorus (33.24 mg·kg^−1^), available potassium (96.12 mg·kg^−1^), effective sulfur (11.6 mg·kg^−1^), and organic matter (18.0 g·kg^−1^), with a pH value of 6.24. The selenium content in the soil was determined to be 0.062 mg/kg.

The plants were planted on 1 October 2023 and 1 October 2024, respectively, in the experimental base of Xuzhou Academy of Agricultural Sciences, Jiangsu Province, China, and harvested on 20 May 2024 and 20 May 2025, respectively. After harvest, the bulbs were spread out to dry for three weeks.

### 4.2. Experimental Treatments

This experiment used nano-selenium fertilizer (Zhongnong Selenium Science and Technology Rich Selenium Research Institute Co., Ltd., Beijing, China, Se ≥ 5%), EDTA chelated selenium fertilizer (Zhengzhou Zhengyan Biotechnology Co., Ltd., Zhengzhou, China, Se ≥ 10%), organic selenium fertilizer (Yangling Aobang Bioscience Co., Ltd., Xianyang, China, Se ≥ 500 mg·L^−1^), and microbial selenium fertilizer (Zhengzhou Kaijin Agricultural Science and Technology Co., Ltd., Zhengzhou, China, Se ≥ 2000 mg·L^−1^), and designed 13 treatment combinations (Table 6 for details). The tested garlic variety ‘Xusuan918’ (Figure 2) was planted in two seasons on 1 October 2023 and 2024, respectively, in the experimental base of Xuzhou Academy of Agricultural Sciences (Xuzhou, China), using a randomized block design with three replicates per treatment. The planting specifications were 12 cm between plants and 20 cm between rows, with a plot area of 10 m^2^ (2 m × 5 m). Field management was carried out according to conventional measures.

The application of selenium (Se) fertilizer was conducted via foliar application during the critical growth stages of garlic, specifically during the regreening stage (March 20, 2024 and 2025), the bolting stage (20 April 2024 and 2025), and the bulb enlargement stage (30 April 2024 and 2025). Each critical growth stage received a single foliar application, with 2 L of selenium fertilizer applied per plot, and the specific concentrations of the treatments are detailed in Table 6. Seven days after each treatment, functional leaves were collected, quickly frozen in liquid nitrogen, and stored at −80 °C in a Thermo Scientific ULT Freezer (Waltham, MA, USA). The observed indicators included ① At the bolting stage (25 April), plant height and leaf area were measured. ② At the bulb maturity stage, fresh weight and quality parameters (vitamin C, soluble sugar, soluble protein, and allicin content) were measured. ③ After the bulbs were dried, bulb dry weight and agronomic traits (bulb height, bulb diameter, and single bulb weight) were recorded. ④ Selenium content in leaves and bulbs was determined.

### 4.3. Morphological Indicators

This study systematically investigated the morphological indicators from the bolting stage to the bulb harvest stage, including at the bolting stage, plant height (vertical distance from the ground to the highest point in the natural extended state), plant width (maximum horizontal diameter of the canopy), leaf length (natural length from the base to the tip), leaf width (vertical distance at the widest part of the leaf), pseudostem height (height from the ground to the base of the first leaf sheath), pseudostem diameter (diameter 2 cm above the ground), and the number of leaves per plant; at the bulb harvest stage, bulb height (vertical distance from the base to the top bud), bulb diameter (maximum equatorial diameter), and single bulb weight (net weight after removing roots and leaves) (Figure 2b). All measurements were conducted according to the standards of “Descriptive Specifications and Data Standards for Garlic Germplasm Resources” by Li Xixiang [57], using a digital vernier caliper (accuracy 0.01 mm), a ruler (accuracy 1 mm), and an electronic balance (accuracy 0.01 g). Ten representative plants were randomly selected from each treatment, and the measurements were repeated three times. The measurements at the bolting stage were taken when the flower stem extended 3 cm out of the leaf sheath, and the measurements at the bulb harvest stage were completed within 24 h after physiological maturity.

### 4.4. Bulb Yield Indicators

This study used a standard yield measurement method to determine the yield of garlic bulbs: three representative rows of garlic were selected from each experimental plot as the sampling area (total area 6 m^2^). Fresh weight (net weight of bulbs after removing leaves and roots) was immediately measured after harvest, and then the samples were dried for three weeks, and dry weight (net weight after removing roots and leaves) was measured (Figure 2b). All weight data were obtained using a standard electronic scale (accuracy 0.01 kg), and the final yield data were converted to yield per hectare based on the sampling area. This yield measurement method ensured the accuracy and comparability of the yield data, providing a reliable basis for subsequent analysis.

### 4.5. Bulb Quality Indicators

After the harvest of fresh garlic (*Allium sativum* L.), three biological replicate samples were randomly selected from each treatment group. The samples were immediately frozen in liquid nitrogen and stored at −80 °C in a freezer (Thermo Scientific ULT Freezer, Waltham, MA, USA) to prevent degradation until analysis.

Allicin content was determined using the method of Feng et al. [58]. Vitamin C (ascorbic acid, ASA) content was measured using an ASA content detection kit (BC1230, Beijing Solarbio Science & Technology Co., Ltd., Beijing, China). Total soluble sugar (TSS) content was determined using a plant soluble sugar content detection kit (BC0035, Beijing Solarbio Science & Technology Co., Ltd., Beijing, China). Total soluble protein (TSP) content was measured by the Bradford method [59], with bovine serum albumin as the standard.

The main instruments and equipment used in the experiment included a grinding instrument (JXFSTPRP-24L, Shanghai Jingxin Industrial Development Co., Ltd., Shanghai, China), a UV spectrophotometer (UV1780, Shimadzu Corporation, Kyoto, Japan), and a refrigerated high-speed centrifuge (Centrifuge 5427 R, Eppendorf AG, Hamburg, Germany).

### 4.6. Determination of Selenium Content

Weigh 0.2 g to 0.8 g (accurate to 0.001 g) of solid sample and place it in a digestion tube. Add 10 mL of HNO_3_ and 2 mL of H_2_O_2_, shake well to mix. Digest in a microwave digestion instrument (MWD-500, Shanghai Metash Instruments Co., Ltd., Shanghai, China). The recommended microwave digestion conditions are shown in Table S3. After digestion, allow to cool and transfer the digestion tube to an acid evaporation instrument to continue heating until nearly dry, but do not evaporate to dryness. Then, add 5 mL of HCl solution (6 mol∙L^−1^) and continue heating until the solution becomes clear and colorless with white smoke. Cool and transfer to a 10 mL volumetric flask. Add 2.5 mL of potassium ferricyanide solution (100∙gL^−1^) and make up to volume with water, mix well, and stand for testing. At the same time, conduct a reagent blank test. All samples are then tested on the instrument (AFS-8220, Titan Instruments, Beijing, China).

### 4.7. Comprehensive Evaluation

#### 4.7.1. Data Standardization

Firstly, the morphological characteristics of the aboveground parts of the plants, bulb yield, nutritional components of the bulbs, and selenium content of the bulbs obtained from each treatment in the experiment were standardized. A value β was defined as the standard value of each index. The specific values are shown in Table S1.

β = Measured value of each treatment/Measured value of the control (1)

#### 4.7.2. Principal Component Scores

The principal component scores were calculated based on the loadings of each principal component on the various indices and the β values of each index according to Formula (1):

ZKi = M1 * β1 + M2 * β2 + …… + M18 * β18(2)

where Zki represents the score of the i-th principal component for the k-th treatment, k ∈ (1, 13), i ∈ (1, 4), Mn represents the loading of the i-th principal component on the n-th index, n ∈ (1, 18), and βn represents the β value of the corresponding index.

#### 4.7.3. Weights of Each Principal Component

The weight represents the proportion of the contribution rate of each principal component to the cumulative contribution rate of the four principal components. It is calculated according to Formula (3):

(3) Ti = Pi/∑Pi   i∈(1, 4)

#### 4.7.4. Membership Function Values

The membership function values of each index for each principal component were calculated based on the scores of each principal component according to Formula (4):

(4) $$\mu (\mathrm{Zki})\;=\;\frac{Z\mathrm{ki}-Z\min}{Z\max-Z\min}$$

where Zki represents the score of the i-th principal component for the k-th treatment, Zmax represents the maximum score of the i-th principal component, and Zmin represents the minimum score of the i-th principal component. The ranges of i and n are the same as in Formula (3).

#### 4.7.5. Evaluation of Fertilization Effects for Each Treatment

To compare the strength of the fertilization effects, a value D was defined to measure the strength of the fertilization effect and was calculated according to Formula (5):

(5) $$Dk=\sum_{1}^{4}Ti*\mu Z\mathrm{ki}$$

The ranges of values for k and i are the same as those mentioned earlier.

### 4.8. Data Processing and Analysis

Data analysis in this study was conducted using SPSS v22.0 (IBM, Chicago, IL, USA), and the Duncan Multiple Range Test (DMRT) was employed to evaluate the significant differences among different treatments. The threshold for statistical significance was set at *p* < 0.05.

Principal component analysis (PCA) and image generation were conducted using GraphPad Prism 9.5.0 (GraphPad Software, San Diego, CA, USA).

## 5. Conclusions

This study conducted a systematic evaluation of four selenium fertilizer types (nano-Se, EDTA-chelated Se, organic Se, and microbial Se) applied through foliar spraying at three concentration levels (50, 25, and 12.5 mg·L^−1^) on garlic cultivation efficacy. Field trials spanning two consecutive production cycles (2023–2025) enabled comprehensive analysis of 12 treatment groups, assessing aboveground morphological traits, bulb yield, selenium accumulation, and nutritional quality, with subsequent evaluation using principal component analysis and membership function methodology. Key findings demonstrate that for Growth Promotion: Selenium fertilization significantly enhanced aboveground growth, with 50 mg·L^−1^ organic Se showing optimal efficacy. Yield enhancement: the 25 mg·L^−1^ microbial Se treatment achieved the most substantial yield increase. Quality improvement: nutritional quality (soluble sugars, vitamin C, etc.) and selenium enrichment were maximized by 50 mg·L^−1^ nano-Se application. Comprehensive evaluation revealed that the 50 mg·L^−1^ nano-Se treatment (D-value: 0.571) exhibited superior performance across all parameters: growth promotion (21.83% increase in plant width), yield improvement (10.04% higher dry weight versus control), and quality enhancement (29-fold increase in bulb selenium content). These results establish this formulation as the recommended fertilization protocol for selenium-enriched garlic production.

## Figures and Tables

**Table 1:** Effects of different treatments on the growth of the aboveground parts of garlic.

Treatment	Plant Height (cm)	Plant Width (cm)	Leaf Length (cm)	Leaf Width (cm)	Number of Functional Leaves per Plant	Height of Above-Ground Pseudostem (cm)	Thickness of Above-Ground Pseudostem (mm)
CK	60.40 ± 5.04 bc	51.10 ± 4.75 d	50.76 ± 3.12 d	3.35 ± 0.20 c	7.03 ± 0.05 a	34.47 ± 0.67 b	15.25 ± 1.37 c
N1	63.14 ± 3.55 abc	62.26 ± 1.72 a	55.80 ± 3.37 abc	3.73 ± 0.39 abc	7.22 ± 0.16 a	38.57 ± 0.88 a	19.37 ± 0.78 a
N2	57.79 ± 6.10 c	56.81 ± 4.19 bc	55.21 ± 3.63 c	3.83 ± 0.36 ab	7.17 ± 0.19 a	36.91 ± 1.83 a	17.14 ± 1.34 b
N3	65.35 ± 1.66 ab	60.59 ± 1.18 abc	59.07 ± 0.97 ab	3.69 ± 0.32 abc	7.31 ± 0.47 a	38.15 ± 0.46 a	18.48 ± 0.44 ab
E1	66.03 ± 4.79 ab	62.15 ± 1.91 a	59.23 ± 1.26 ab	3.79 ± 0.38 abc	7.18 ± 0.33 a	36.32 ± 3.15 ab	18.22 ± 1.01 ab
E2	65.04 ± 4.24 ab	60.37 ± 2.77 abc	55.64 ± 3.58 bcd	3.61 ± 0.30 abc	7.08 ± 0.17 a	37.18 ± 1.63 a	17.69 ± 0.83 ab
E3	67.65 ± 4.16 a	61.83 ± 2.77 a	58.95 ± 1.65 ab	3.53 ± 0.10 abc	7.17 ± 0.34 a	37.63 ± 1.81 a	18.40 ± 1.14 ab
O1	64.79 ± 2.80 ab	63.45 ± 1.90 a	60.15 ± 2.52 a	3.88 ± 0.40 a	7.38 ± 0.34 a	38.93 ± 1.05 a	18.62 ± 1.54 ab
O2	61.17 ± 3.25 abc	55.42 ± 4.53 d	52.95 ± 4.67 d	3.40 ± 0.09 bc	7.05 ± 0.10 a	37.48 ± 0.83 a	19.48 ± 1.93 a
O3	62.78 ± 4.95 abc	56.64 ± 4.02 c	57.49 ± 2.20 ab	3.51 ± 0.14 abc	7.10 ± 0.20 a	38.36 ± 1.45 a	17.71 ± 0.91 ab
M1	65.38 ± 3.94 ab	60.43 ± 1.66 abc	59.22 ± 1.03 ab	3.64 ± 0.22 abc	7.05 ± 0.10 a	37.19 ± 1.27 a	19.28 ± 0.75 a
M2	63.08 ± 3.69 abc	58.68 ± 3.42 abc	59.53 ± 1.53 ab	3.65 ± 0.14 abc	7.07 ± 0.14 a	37.58 ± 1.87 a	18.25 ± 0.37 ab
M3	64.92 ± 3.21 ab	58.33 ± 3.56 abc	59.50 ± 2.35 ab	3.50 ± 0.23 abc	7.11 ± 0.16 a	37.38 ± 1.86 a	19.29 ± 1.09 a

CK, blank control; N, Selenium Nano-fertilizer; E, EDTA-Se Chelated Fertilizer; O, Organic Selenium Fertilizer; M, Microbial Selenium Fertilizer. Values (a,b) represent mean ± SE. Ten representative plants were randomly selected for each treatment, with the experiment repeated three times. Significant differences were analyzed using Duncan’s multiple range test. Lowercase letters indicate significant differences between treatments (*p* < 0.05) in this paper.

**Table 2:** Yield of garlic bulbs under different treatments.

Treatment	Fresh Weight (t ha^−1^)	Dry Weight (t ha^−1^)	DW/FW Ratio (%)	Bulb Height (mm)	Bulb Width (mm)	Single Bulb Weight (g)
CK	24.10 ± 1.29 e	17.64 ± 0.21 c	73.18 ± 2.21 c	35.42 ± 0.77 b	56.80 ± 2.99 c	60.63 ± 1.53 d
N1	25.07 ± 0.53 cde	19.40 ± 0.43 ab	77.39 ± 1.40 ab	38.07 ± 1.38 a	62.02 ± 1.58 ab	68.91 ± 2.71 ab
N2	23.29 ± 1.01 e	18.61 ± 0.47 bc	79.91 ± 2.62 a	35.84 ± 1.28 b	59.59 ± 1.38 b	63.87 ± 2.05 cd
N3	26.66 ± 1.40 abcd	19.89 ± 1.27 ab	74.58 ± 5.96 bc	36.90 ± 0.65 ab	61.55 ± 2.49 ab	67.08 ± 1.99 abc
E1	25.30 ± 1.83 bcde	19.50 ± 1.73 ab	77.07 ± 1.77 ab	35.93 ± 2.15 b	60.06 ± 2.12 ab	68.26 ± 2.85 abc
E2	25.82 ± 2.00 abcd	19.59 ± 1.16 ab	75.86 ± 2.77 bc	35.92 ± 1.11 b	60.51 ± 1.42 ab	67.44 ± 2.21 abc
E3	25.84 ± 2.30 abcd	19.49 ± 1.41 ab	75.45 ± 1.88 bc	36.96 ± 0.28 ab	62.83 ± 0.69 ab	67.81 ± 2.19 abc
O1	26.45 ± 1.56 abcd	19.72 ± 0.89 ab	74.58 ± 0.78 bc	36.80 ± 0.54 ab	61.73 ± 1.30 ab	66.57 ± 2.51 bc
O2	24.81 ± 0.17 cde	18.99 ± 0.31 bc	76.55 ± 1.79 ab	36.61 ± 1.06 ab	61.60 ± 1.74 ab	65.37 ± 3.54 bc
O3	26.89 ± 2.16 abc	20.34 ± 1.58 ab	75.64 ± 1.48 bc	37.20 ± 0.74 ab	63.29 ± 1.36 a	68.94 ± 2.47 ab
M1	27.11 ± 1.15 abc	20.20 ± 0.57 ab	74.51 ± 1.39 bc	36.95 ± 1.41 ab	61.29 ± 3.03 ab	69.21 ± 6.07 ab
M2	28.39 ± 2.05 a	20.95 ± 1.61 a	73.79 ± 2.82 c	36.03 ± 0.95 ab	60.97 ± 1.72 ab	71.53 ± 2.59 a
M3	27.73 ± 1.15 ab	20.32 ± 0.86 ab	73.27 ± 1.54 c	36.42 ± 1.21 ab	61.06 ± 1.72 ab	69.57 ± 2.81 ab

The ratio of dry fresh weight of garlic bulbs is the ratio of the average dry fresh weight of bulbs. Values (a,b) represent mean ± SE. The fresh and dry weights of the bulbs were determined once for each experimental plot, with three replications. For each treatment, 10 bulbs were sampled to measure the relevant parameters of individual bulbs, with three independent replicates conducted for each treatment. Significant differences were analyzed using Duncan’s multiple range test. Lowercase letters indicate significant differences between treatments (*p* < 0.05) in this paper.

**Table 3:** Nutritional content of garlic bulb yield under different treatments.

Treatment	Allicin (mg g^−1^)	Vc (mg g^−1^)	TSS (%)	TSP (mg g^−1^)
CK	2.41 ± 0.08 e	0.86 ± 0.01 cd	17.21 ± 0.22 e	7.30 ± 0.08 e
N1	2.70 ± 0.09 a	0.93 ± 0.02 a	19.35 ± 0.24 a	7.72 ± 0.03 a
N2	2.61 ± 0.05 ab	0.91 ± 0.03 ab	19.00 ± 0.11 ab	7.68 ± 0.07 b
N3	2.54 ± 0.01 bcd	0.88 ± 0.01 ab	18.95 ± 0.18 ab	7.62 ± 0.04 b
E1	2.48 ± 0.10 cde	0.87 ± 0.02 bcd	18.65 ± 0.39 bc	7.48 ± 0.05 c
E2	2.48 ± 0.06 cde	0.85 ± 0.03 cd	18.34 ± 0.19 c	7.62 ± 0.02 b
E3	2.49 ± 0.04 cde	0.85 ± 0.03 cd	18.36 ± 0.31 c	7.35 ± 0.04 de
O1	2.59 ± 0.03 abc	0.83 ± 0.02 d	19.46 ± 0.05 a	7.73 ± 0.04 a
O2	2.62 ± 0.12 ab	0.86 ± 0.03 bcd	18.90 ± 0.06 ab	7.62 ± 0.04 b
O3	2.53 ± 0.06 bcd	0.83 ± 0.02 d	18.87 ± 0.11 ab	7.43 ± 0.01 cd
M1	2.54 ± 0.02 bcd	0.85 ± 0.03 cd	18.58 ± 0.34 bc	7.41 ± 0.06 cd
M2	2.44 ± 0.01 de	0.87 ± 0.04 bcd	17.80 ± 0.48 d	7.38 ± 0.06 d
M3	2.43 ± 0.10 de	0.87 ± 0.02 bcd	17.48 ± 0.35 de	7.39 ± 0.04 d

Vc, Vitamin C; TSS, Total soluble sugar; TSP, Total soluble protein. Values (a,b) represent mean ± SE. For each treatment, measure three bulb-related indicators with three replicates. Significant differences were analyzed using Duncan’s multiple range test. Lowercase letters indicate significant differences between treatments (*p* < 0.05) in this paper.

**Table 4:** Selenium content in garlic with different treatments.

Treatment	Selenium Content in Leaves (mg kg^−1^)	Selenium Content in Bulbs (mg kg^−1^)	Bulb-to-Leaf Ratio (%)
CK	0.02 ± 0.02 e	0.01 ± 0.01 e	50.32 ± 3.16 c
N1	0.50 ± 0.10 a	0.29 ± 0.05 a	58.48 ± 5.14 ab
N2	0.29 ± 0.06 b	0.16 ± 0.04 b	55.25 ± 5.38 b
N3	0.29 ± 0.11 b	0.12 ± 0.06 bc	41.33 ± 3.94 c
E1	0.15 ± 0.06 cde	0.09 ± 0.02 bcd	60.78 ± 5.71 a
E2	0.11 ± 0.04 cde	0.05 ± 0.02 de	45.24 ± 4.28 c
E3	0.08 ± 0.05 cde	0.04 ± 0.01 de	50.33 ± 3.78 bc
O1	0.22 ± 0.07 bc	0.10 ± 0.04 bcd	45.44 ± 5.48 c
O2	0.21 ± 0.02 bcd	0.09 ± 0.02 bcd	43.22 ± 1.33 c
O3	0.18 ± 0.10 cd	0.09 ± 0.03 bcd	50.65 ± 6.64 bc
M1	0.13 ± 0.04 cde	0.08 ± 0.03 cde	62.89 ± 1.46 a
M2	0.08 ± 0.02 de	0.05 ± 0.01 cde	63.47 ± 4.69 a
M3	0.06 ± 0.01 de	0.03 ± 0.01 de	50.74 ± 2.30 bc

The stem-to-leaf ratio is the ratio of the average selenium content in garlic bulbs and leaves of different treatments. Values (a,b) represent mean ± SE. For each treatment, measure three bulb-related indicators with three replicates. Significant differences were analyzed using Duncan’s multiple range test. Lowercase letters indicate significant differences between treatments (*p* < 0.05) in this paper.

**Table 5:** Principal Component Score (Z), Membership Function Value (μ), Weight, Comprehensive Evaluation Value (D), and Ranking Under Different Treatments.

Treatment	Z (1)	Z (2)	Z (3)	Z (4)	μ (1)	μ (2)	μ (3)	μ (4)	D	Ranking
Ck	11.578	0.385	0.187	0.733	0	1	1	0	0.429	13
N1	48.188	−34.172	−4.552	11.291	1	0	0	1	0.571	1
N2	31.671	−18.704	−2.333	6.486	0.549	0.448	0.468	0.545	0.507	2
N3	29.386	−15.605	−1.989	5.453	0.486	0.537	0.541	0.447	0.506	3
E1	22.375	−8.897	−1.01	3.754	0.295	0.731	0.747	0.286	0.483	5
E2	18.186	−4.939	−0.538	2.41	0.181	0.846	0.847	0.159	0.464	10
E3	16.633	−3.138	−0.332	1.933	0.138	0.898	0.89	0.114	0.461	11
O1	25.761	−11.931	−1.427	4.413	0.387	0.644	0.659	0.349	0.496	4
O2	24.298	−11.169	−1.534	4.09	0.347	0.666	0.637	0.318	0.479	7
O3	23.397	−9.871	−1.323	3.906	0.323	0.703	0.681	0.301	0.482	6
M1	21.106	−7.446	−0.948	3.362	0.26	0.773	0.761	0.249	0.478	8
M2	17.265	−3.676	−0.441	2.319	0.155	0.882	0.868	0.15	0.465	9
M3	15.204	−1.702	−0.205	1.641	0.099	0.94	0.917	0.086	0.456	12
Weight	0.4986	0.3259	0.1028	0.0726						

**Table 6:** Types and concentrations of selenium fertilizers for different treatments.

Types of Fertilizers	Treatment	Selenium Concentration (mg·L^−1^)
	N1	50
Nano-Se	N2	25
	N3	12.5
	E1	50
EDTA-Se	E2	25
	E3	12.5
	O1	50
Organic-Se	O2	25
	O3	12.5
	M1	50
Microbial-Se	M2	25
	M3	12.5
	CK	0

## Data Availability

The data presented in this study are available on request from the corresponding author due to the project involved in the current data has not yet been completed.

## Supplementary Materials

The following supporting information can be downloaded at: https://www.mdpi.com/article/10.3390/plants14162505/s1, Table S1: β value; Table S2: Correlation matrix; Table S3: Microwave Digestion Temperature Rise Program; Table S4: Principal component coefficients, contribution rates and principal component load matrices of different treatments; Table S5: PCA analysis data; Table S6: Weather Conditions from February to May 2022–2023.
