# Supplementary material for: The Effect of Foliar Spraying of Different Selenium Fertilizers on the Growth, Yield, and Quality of Garlic (Allium sativum L.)
Source: Plants (Basel). 2025 Aug 12;14(16):2505. doi: 10.3390/plants14162505 (PMC12389049; doi:10.3390/plants14162505)
Supplement: Supplementary file 1 [file plants-14-02505-s001.zip › Table S3 Microwave Digestion Temperature Rise Program.pdf]

Table S3 Microwave digestion temperature rise program

Step	Temperature (℃)	Heating time (min)	Constant temperature time(min)
1	120	6	1
2	150	3	5
3	200	5	10
