# Supplementary material for: The Effect of Foliar Spraying of Different Selenium Fertilizers on the Growth, Yield, and Quality of Garlic (Allium sativum L.)
Source: Plants (Basel). 2025 Aug 12;14(16):2505. doi: 10.3390/plants14162505 (PMC12389049; doi:10.3390/plants14162505)
Supplement: Supplementary file 1 [file plants-14-02505-s001.zip › Table S4 Principal component coefficients, contribution rates and principal component load matrices of different treatments..pdf]

Table S4. Principal component coefficients, contribution rates and principal component load matrices of different treatments.

	Principal component factor			
	1	2	3	4
Fresh weight	0.319	0.85	-0.116	0.161
Dry weight	0.565	0.73	-0.103	0.245
Plant height	0.406	0.67	0.201	-0.2
Plant width	0.821	0.25	0.405	-0.01
Leaf length	0.612	0.66	0.347	0.125
Leaf width	0.642	-0.18	0.66	0.165
Number of functional leaves per plant	0.668	-0.18	0.553	-0.196
Height of above-ground pseudostem	0.912	0.09	-0.176	-0.177
Thickness of above-ground pseudostem	0.742	0.26	-0.329	0.12
Bulb height	0.789	0.00	-0.449	-0.103
Bulb width	0.794	0.33	-0.304	-0.289
Single bulb weight	0.621	0.65	-0.164	0.361
Allicin	0.647	-0.69	-0.265	-0.094
Vc	0.21	-0.643	0.003	0.678
TSS	0.788	-0.418	0.011	-0.349
TSP	0.677	-0.643	0.095	-0.098
Selenium content in leaves	0.698	-0.675	-0.091	0.135
Selenium content in bulbs	0.667	-0.663	-0.091	0.259
Characteristic value	7.988	5.221	1.648	1.163
Contribution rate(%)	44.379	29.007	9.153	6.46
Cumulative contribution rate%	44.379	73.386	82.539	88.999

The abbreviations are the same as the previous table.
