# Supplementary material for: The Effect of Foliar Spraying of Different Selenium Fertilizers on the Growth, Yield, and Quality of Garlic (Allium sativum L.)
Source: Plants (Basel). 2025 Aug 12;14(16):2505. doi: 10.3390/plants14162505 (PMC12389049; doi:10.3390/plants14162505)
Supplement: Supplementary file 1 [file plants-14-02505-s001.zip › Table S6 Weather Conditions from February to May 2022-2023.pdf]

Year	Month	Average maximum temperatur （℃）	Average minimum temperature （℃）	Rainfall （mm）
2023		9	0	79.5
2024	2	8	-2	493.5
2025		10	-1	2
2023		18	6	150.9
2024	3	16	6	15.3
2025		18	6	11.7
2023		21	10	124.2
2024	4	24	12	13.4
2025		27	12	0
2023		26	16	160.8
2024	5	29	16	0.6
2025		30	17	13.8
